# Cancer-related hypercalcemia and potential treatments

**DOI:** 10.3389/fendo.2023.1039490

**Published:** 2023-03-22

**Authors:** Elvina Almuradova, Irfan Cicin

**Affiliations:** ^1^ Tınaztepe Galen Hospital, Medical Oncology Center, Izmir, Türkiye; ^2^ Medical Oncology Department, Faculty of Medicine, Trakya University, Trakya, Türkiye

**Keywords:** cancer, hypercalcaemia, treatment, maligancy, therapy

## Abstract

Cancer-related hypercalcemia is a common finding typically seen in patients with advanced cancer and occurs in about 20 to 30 percent of cases. The most common cause of hypercalcemia in hospitalized patients is hypercalcemia due to malignancy.This clinical problem is seen in patients with both solid tumors and patients with hematologic malignancies. Hypercalcemia is associated with a poor prognosis in oncology patients. This pathologic condition can occur due to many different mechanisms but is usually caused by abnormal calcium use resulting from bone resorption, intestinal absorption, or renal excretion. Hypercalcemia may present with a wide range of symptoms ranging from gastrointestinal system symptoms to neurologic symptoms. Timely diagnosis and initiation of treatment by the physician significantly reduce the risk of complications. Treatment aims to decrease serum calcium by increasing calciuresis, decreasing bone resorption, and decreasing intestinal calcium absorption. The mainstays of treatment are IV hydration, bisphosphonates and calcitonin, denosumab, and in some patients, prednisone, and cinacalcet. Patients with underlying advanced kidney disease and refractory severe hypercalcemia should be evaluated for hemodialysis. Every physician dealing with oncology patients should know the fastest and most effective management of hypercalcemia. We aimed to contribute in this sense.

## 1 Introduction

Hypercalcemia of malignancy (HCM) is a condition in which the serum calcium level is above normal level (1–3). Symptoms resulting from hypercalcemia can range from mild to life-threatening. Malignancy is one of the most common causes of hypercalcemia, especially in patients with cancer associated with bone metastases (1). It is estimated that hipercalcemia affects aproximately 30% of patients with cancer (3). Common malignancies associated with HCM include multiple myeloma, breast, lung, squamous cell carcinomas, renal, ovarian cancer, and certain lymphomas. The severity of hypercalcemia is categorized according to the serum total calcium level (1). Hypercalcemia is a clinical problem that occurs as a result of abnormal bone formation and resorption process due to cancer. Although hypercalcemia due to malignancy has decreased with the introduction of new treatment agents, it is still a common clinical problem. The aim of this review is to contribute to the awareness of clinicians by summarizing the current literature on the mechanism, diagnosis and management of malignant hypercalcemia.

## 2 Calcium metabolism

**Figure 1:**
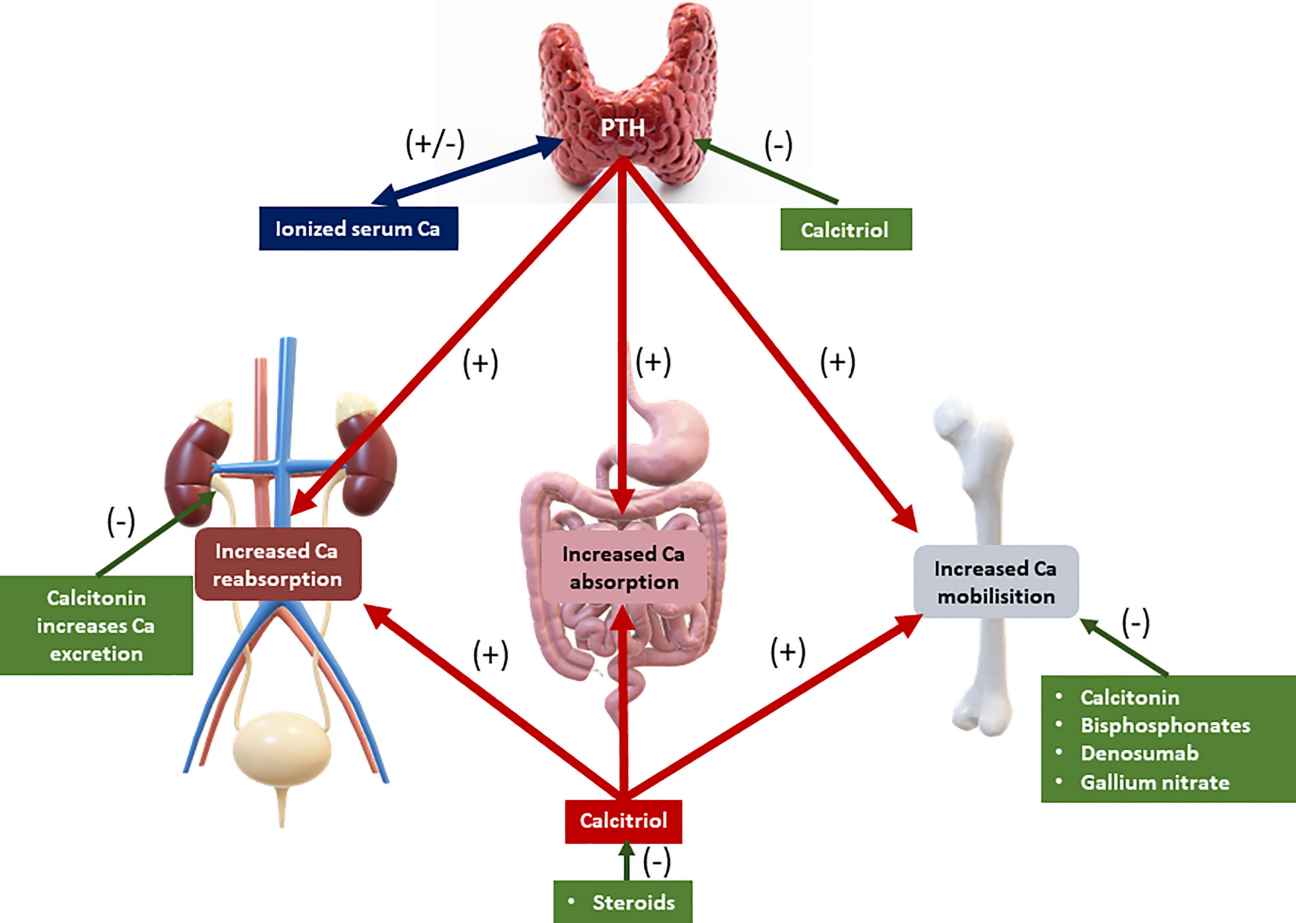
Calcium metabolism. PTH: parathyroid hormone. Green, decreasing effect on serum calcium level. Red: increasing effect on serum calcium level.

**Figure 2:**
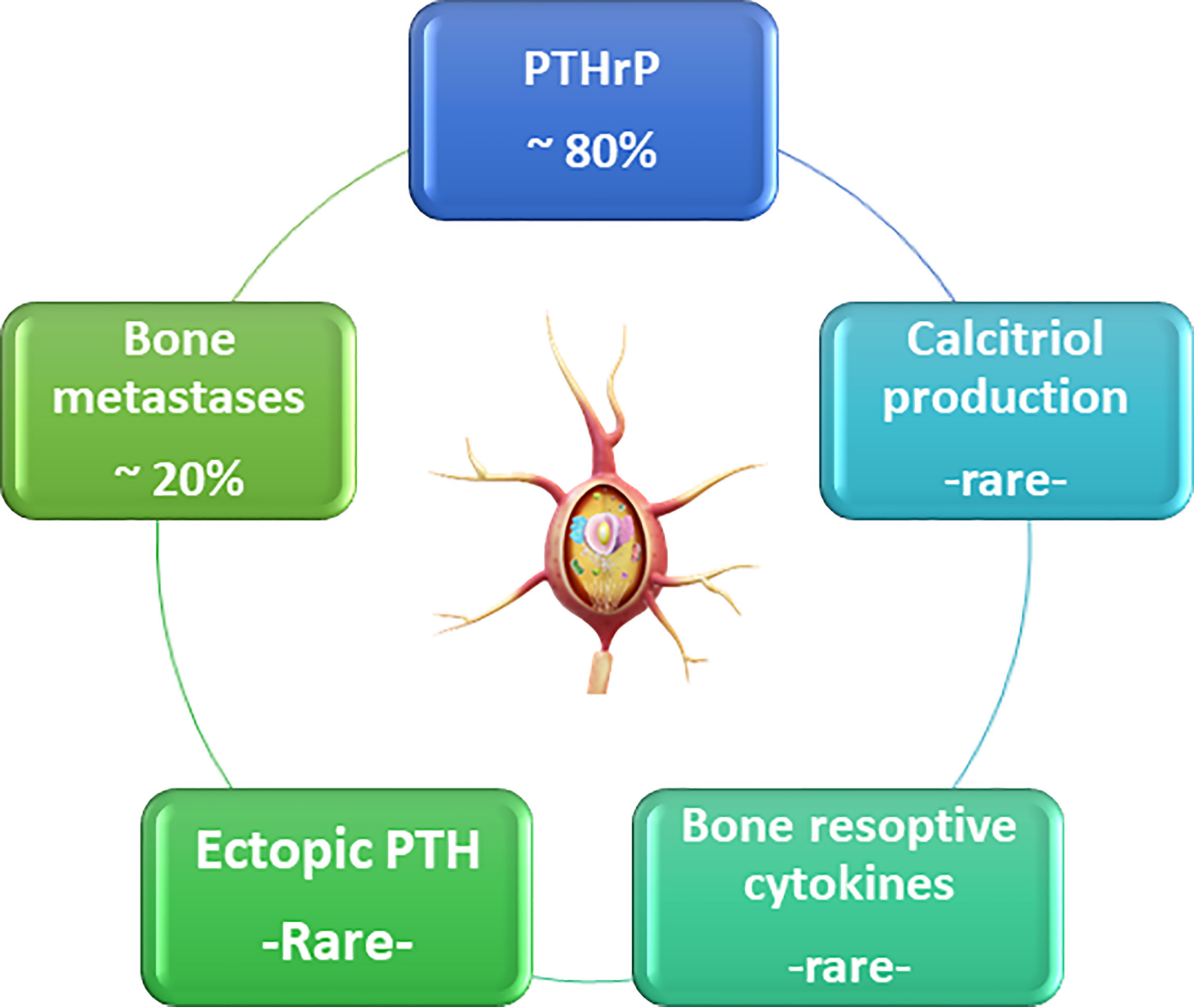
The reasons of malignant hypercalcemia.

Calcium balance refers to the state of calcium stores in the body, especially in the bone. Bone calcium balance can be neutral, positive, or negative, depending on several factors, including growth, aging, and acquired or inherited disorders. Calcium homeostasis refers to the hormonal regulation of ionized serum calcium by parathyroid hormone, 1,25-dihydroxyvitamin D (calcitriol), and serum ionized calcium itself, and these factors together regulate calcium transport in the intestine, kidney, and bone (4–8) (**Figure 1**). Three main mechanisms cause hypercalcemia in malignancies. First, the most common (80%), is the secretion of PTHrP from tumors, which can cause hypercalcemia by acting similarly to parathyroid hormone (PTH) (9). The second is increased calcium absorption by the autonomous production of 1,25(OH)2D by 1α-hydroxylase in the tumor (10). The third mechanism is resorption, which occurs due to the increase in osteoclastic activity of tumor cells in the bone tissue (**Figure 2**) (11).

### 2.1 Parathyroid hormone-related peptide (PTHrP)

The most common cause of hypercalcemia in patients with solid tumors is the secretion of PTHrP (12). This is also known as humoral hypercalcemia of malignancy. This can occur both in solid tumors such as lung, kidney, bladder, breast, head and neck cancers, and in diseases such as non-Hodgkin lymphoma, adult T-cell lymphoma, and chronic myeloid leukemia (13). Hypercalcemia due to PTHrP is frequently observed, especially in tumors with squamous histology (12–14). PTHrP contains homology with PTH, especially according to the sequence of the first 13 amino acids (15). As a result of this close similarity with PTH, PTHrP binds to the same PTH-1 receptor as PTH, thereby activating similar post-receptor pathways. The circulating PTHrP causes stimulation of the PTH receptor in the bone and kidney. This increases bone resorption and increases distal tubular calcium reabsorption, resulting in calcium release from the bone, as well as a decrease in the excretion ability of calcium (14, 15).

The structural difference after the first 13 amino acids of the molecule explains its immunologic difference from PTH. PTHrP is less likely than PTH to stimulate the production of 1,25-dihydroxyvitamin D, so the measurement of 1,25-dihydroxyvitamin D in patients with PTHrP-mediated hypercalcemia may be variable (4, 16). Typical laboratory findings in patients with humoral hypercalcemia are high serum PTHrP and very low or suppressed serum intact PTH and variable serum 1,25-dihydroxyvitamin D levels (16).

### 2.2 1,25(OH)2D Production

In normal individuals, 25-hydroxyvitamin D (calcidiol) is converted to 1,25-dihydroxyvitamin D (calcitriol, the most active form of vitamin D) through 1-hydroxylase in the renal tubules, under the influence of PTH (17). Fibroblast growth factor 23 (FGF-23) inhibits this transformation through hyperphosphatemia (17, 18). Hypercalcemia suppresses the release of PTH and thus the production of 1,25-dihydroxyvitamin D. However, in some tumor types, 25-dihydroxyvitamin D is produced extrarenally from 25-hydroxyvitamin D, independent of PTH control (19).

Uncontrolled production of 1,25-dihydroxyvitamin D (calcitriol) is the cause of almost all cases of hypercalcemia in Hodgkin lymphoma and about one-third of cases of non-Hodgkin lymphoma (20, 21). Hypercalcemia due to this mechanism has also been described in patients with ovarian dysgerminoma and lymphomatoid granulomatosis caused by 1,25-dihydroxyvitamin D (22, 23).

High serum 1,25-dihydroxyvitamin D concentrations may contribute to hypercalcemia by increasing both intestinal calcium absorption and bone resorption. Laboratory evaluation of these patients typically reveals low or suppressed serum PTH and elevated 1,25-dihydroxyvitamin D levels (16).

### 2.3 Bone resorption

Hypercalcemia due to bone resorption occurs as a result of the release of mediators, which increase osteoclastic activity by tumor cells in the bone. The soluble protein RANKL, a member of the TNF family, is a central regulator of osteoclast formation, activity, and survival. This protein is synthesized by osteoblasts and T cells and directs the differentiation and activation of osteoclasts (24). Hematopoietic precursor cells exposed to macrophage colony-stimulating factor (M-CSF) express NFκB (RANK)-binding receptors. When RANK and RANKL combine, they induce differentiation into osteoclastic cells (25).

In addition, multiple RANKL-independent osteoclast (OCL) stimulatory factors, including macrophage-derived protein MIP-1a, ILs 3, -8, -6, -17, -18, and Activin A, are produced or induced by cancer cells (5). MIP-1α is a chemokine produced by multiple myeloma (MM) cells in 70% of patients and is a potent inducer of human OCL formation (26). MIP-1α gene expression is highly associated with bone resorption in MM, and high MIP-1α levels are associated with an extremely poor prognosis. MIP-1α acts as a chemotactic factor for OCL precursors and can induce differentiation of OCL progenitors, contributing to RANKL-independent OCL formation (27). In addition, MIP-1α potentiates both RANKL and interleukin (IL)-6-induced OCL formation (28). MIP-1a also increases the expression of β1 integrins on tumor cells, allowing them to settle in the bone marrow. This results in increased production of RANKL, IL-6, vascular endothelial growth factor (VEGF), and tumor necrosis factor-a (TNF-α) by bone marrow stromal cells, further enhancing tumor cell growth, angiogenesis, and bone resorption (29). IL-3 is another OCL-stimulating factor found in the bone marrow. IL-3 can also indirectly induce osteoclastogenesis by increasing the effects of RANKL on the development of OCLs (30). This factor also contributes to bone resorption by stimulating Activin A, which prevents osteoblast differentiation (31). TNF-α is a bifunctional cytokine that can induce OCL formation and suppress OB differentiation through its effects on Runx2 and Gfi-1 expression in bone marrow stromal cells (32). Transforming growth factor (TGF-β) is an upregulated factor in bone metastasis that has multiple effects on the tumor-bone microenvironment. TGF-β can increase the production of IL-6 and VEGF by tumor cells (33).

Apart from these three main mechanisms, there are other less common causes of hypercalcemia due to malignancy.

### 2.4 Ectopic PTH secretion

Tumors secreting ectopic PTH have been reported as case reports in the literature (34). Examples of these tumor types are ovarian carcinoma, small cell and squamous cell lung carcinomas, neuroectodermal tumors, thyroid papillary carcinoma, metastatic rhabdomyosarcoma, pancreatic malignancy, and gastric carcinoma (35). Laboratory analyses of these patients typically show elevated PTH, calcium, and low phosphorus levels (36).

### 2.5 Pseudohypercalcemia

Another rare cause of hypercalcemia is pseudohypercalcemia. This is due to measurement errors due to calcium binding to an abnormal immunoglobulin. It is possible to distinguish this using atomic absorption spectrophotometry (37).

### 2.6 The role of microRNAs (miRNAs)

The miRNAs act as subtle modulators in maintaining bone homeostasis. Basic evidence that miRNAs are essential for osteoclastogenesis is provided by genetic studies that delete DICER1, an enzyme essential for their biogenesis (38). DICER-deficient mice and osteoclast-specific DICER gene deficiency lead to impairment in both OC formation and activity (38). It is known that there are miRNAs that support and suppress the formation of OCs (39). Although the functions of a few of them are known, with increasing studies in the near future, miRNAs will also be used in the treatment of problems such as malignancy and hypercalcemia.

## 3 Clinical findings

Symptoms of hypercalcemia depend on at least two factors: the degree of hypercalcemia and the rate of change in serum calcium. Degrees of hypercalcemia relative to serum total calcium level are as follows: mild hypercalcemia, 10.5 to 11.9 mg/dL; moderate hypercalcemia, 12 to 13.9 mg/dL; and severe hypercalcemia, 14 mg/dL or above (40). Mild hypercalcemia may be asymptomatic or associated with mild nonspecific symptoms such as numbness and pain (41). In contrast, severe, rapidly progressive hypercalcemia can be associated with a variety of life-threatening symptoms (41, 42).

It is important to note that at least two factors, such as the degree of hypercalcemia and the rate of its development, play a role in the development of clinically overt hypercalcemia. Patients with malignant hypercalcemia develop hypercalcemia at a very high level and in a short time, and therefore they are more symptomatic than patients with other causes of hypercalcemia (40). The major systems affected by hypercalcemia are neuropsychiatric, gastrointestinal, and renal systems (43–45). Almost all affected individuals have GI symptoms. Mild calcium elevation may manifest as anorexia and constipation. Nausea and vomiting may develop in patients with severe hypercalcemia, but these conditions can easily be confused with the adverse effects of tumor treatment or symptoms directly produced by the tumor itself (3, 45). Cramping abdominal pain, such as those seen in people with primary hyperparathyroidism, is rarely encountered, but severe outcomes such as peptic ulceration and pancreatitis are much less common in malignant hypercalcemia (46).

Hypercalcemia impairs the concentration ability of the kidney (47). Tubular damage causes acquired renal tubular acidosis, glycosuria, and aminoaciduria (47). Renal manifestations consist of nephrogenic diabetes insipidus resulting in polyuria. All patients with clinically overt hypercalcemia have volume depletion resulting in an increase in creatinine level and a decrease in glomerular filtration rate, explained by a decrease in oral intake due to polyuria and nausea and vomiting (47, 48). Nephrocalcinosis and nephrolithiasis require long-term hypercalcemia and therefore are not common in malignant hypercalcemia (49).

Neuropsychiatric symptoms such as apathy, mood changes, and fatigue are often seen as symptoms of hypercalcemia, which can be overlooked and can be attributed to the underlying neoplasm. In oncology patients, muscle strength defect leads to mobility restriction, which leads to more calcium resorption from the bone and increases hypercalcemia (50). As hypercalcemia continues to worsen, severe symptomatology may occur, including changes in mental status, confusion, and eventually coma (50, 51). Rarely, patients may even develop posterior reversible leukoencephalopathy syndrome (PRES), which is manifested by headaches, seizures, and imaging findings of subcortical edema (52).

Cardiovascular system findings are seen as a shortening of the QT interval on electrocardiograms (53). Malignant ventricular arrhythmias such as ventricular fibrillation may develop in patients with severe hypercalcemia (54).

Bone pain is a common symptom, both due to the malignancy itself and hypercalcemia. Bone pain may be associated with increased intramedullary pressure, ischemia, or the presence of metastases within the bone causing areas of microfracture, but the symptom is also present in the absence of demonstrable metastatic disease (55).

## 4 Evaluation of patients

The first evaluation of patients with clinical signs of hypercalcemia starts with serum calcium measurement. Serum calcium is the sum of the physiologically inactive carrier-bound calcium and the active form of serum calcium, ionized calcium (56). Therefore, if the calcium level is found to be high in the analysis, it is necessary to know whether these tests measure ionized calcium or total calcium. Total calcium levels can be affected by many factors such as serum protein and pH. In such cases, the value of ionized calcium is more important (57). Serum albumin measurements are necessary for the interpretation of serum calcium levels because calcium homeostasis is greatly affected by albumin concentrations. If albumin is abnormal, serum calcium should be corrected using the following formula (58):

Corrected calcium = Total calcium + [0.8 × (4.0 – albumin)]

Protein levels and thus calcium levels may be elevated in patients with severe dehidratation (57). It is important to remember that the albumin-calcium system is highly sensitive to pH and changes in pH can alter the fraction of albumin-bound calcium ions (59). Therefore, it is always recommended to confirm high serum calcium with a repeat test (60).

The next step in the evaluation of a patient with malignancy-related hypercalcemia consists of measuring both PTH and PTHrP. PTH and PTHrP are similar molecules; therefore, both will not rise at the same time unless there is more than one cause. In most cases of malignancy, serum PTH levels appear to be suppressed or normal (10). High-normal PTH levels in the setting of hypercalcemia suggest the presence of PTH-mediated hypercalcemia or parathyroid carcinoma (61). Serum phosphorus and other electrolytes should be measured, as hypercalcemia due to PTHrP or PTH may cause hypophosphatemia, hyperchloremia, and mild metabolic alkalosis (62). If PTHrP levels are low, the next step should include measuring 1,25-dihydroxyvitamin D levels to screen for vitamin D-mediated hypercalcemia. In patients with low PTH, PTHrp, and 1,25-dihydroxyvitamin D, hypercalcemia due to osteolytic metastases may be considered the cause of malignancy-associated hypercalcemia (61).

Although rare, patients may have familial hypercalcemia symptoms together with malignancy. The 24-hour urinary calcium clearance-creatinine clearance ratio (FeCa) may be valuable for the assessment of familial hypocalciuric hypercalcemia (63). If FeCa is low (less than 0.01), familial hypocalciuric hypercalcemia should be suspected and definitive evaluation may include genetic testing for mutations in the *CASR*, *AP2S1*, or *GNA11* genes (64). However, it is important to note that more than one malignancy-associated hypercalcemia mechanism can be seen in oncology patients.

### 4.1 Treatment of hypercalcemia

The main goal in the treatment of hypercalcemia is to find the underlying cause and initiate treatment (**Table 1**). Although all attention can be given to this goal, especially in mild hypercalcemia, moderate-to-severe hypercalcemia also requires concomitant symptomatic treatment. Patients with a calcium value of >14 mg/dL (>3.5 mmol/L) require more aggressive treatment. In addition, patients with neurologic symptoms (eg, lethargy, stupor), regardless of serum calcium levels, require urgent aggressive treatment (65, 66).

**Table 1:** Treatment options for hypercalcemia of malignancy.

Agent	Regimen	Mechanism of action	Onset	Duration	Side Effects
**0.9% saline**	2-4 l/day or 200-500 ml/h	Enhance renal excretion of Ca^2+^	Immediate	1-3 days (depends on cardiovascular and renal status)	Volume overload
**Zoledronic acid** **or** **Pamidronate**	4 mg IV over 15 to 30 minutes in a solution of 50-100 ml NS or D5W 60 to 90 mg IV over 4 to 24 hours	Inhibits osteoclastic bone resorption	48 hours	Every 3-4 weeks May be additional	Renal toxicity, acute-phase reactions, gastrointestinal toxicity, hypocalcemia and osteonecrosis of the jaw
**Denosumab**	120 mg SQ	İnhibits the binding of RANKL with its receptor RANK and decreases OC activity	7-10 days	Every 4 weeks and additional on days 8 and 15 for first month	Allergic reactions, hypocalcemia, osteonecrosis
**Calcitonin**	4 units/kg SQ repeated every 6-12 hours	Increases renal calcium excretion reduce bone resorption by interfering with OC function	4-6 hours	24 to 48 hours	Pain at the injection site and cutaneous flushing, anaphylactic reactions
**Glucocorticoids**	200-400 mg/day of hydrocortisone 10-20 mg/day of prednisone	Inhibit 1,25(OH)_2_ D synthesis and thus calcium absorption from the intestine	7 days	3-10 days (unclear)	Myopathy, immunosuppression, elevated blood glucose
**Gallium Nitrate**	100 to 200 mg/m2 IV over 24 hours for 5 days	inhibits osteoclast activity	4 days	2 weeks	Nephrotoxicity, bone marrow supression

Ca^2+^ calcium ions; SQ subcutaneously; D5W 5% dextrose in water; NS normal saline; OC osteoclastic; RANK receptor activator of nuclear factor kappa-B ligand.

### 4.1 Restoration of intravascular volume and promotion of renal calcium excretion

As mentioned before, patients with hypercalcemia present with nausea, vomiting, inadequate hydration due to altered mental status, and a dehydrated state due to nephrogenic diabetes insipidus caused by hypercalcemia (47, 67). In addition, the volume reduction itself also reduces the renal clearance of calcium due to increased calcium reabsorption due to hypovolemia in the kidneys, resulting in a vicious circle. Therefore, hydration is one of the cornerstones of treatment in hypercalcemia. Isotonic crystalloid solutions (e.g. normal saline) should be used for IV hydration. Typically, patients with advanced hypercalcemia should start at a rate of approximately 200-300 mL/hour (68). Patients should be evaluated periodically for signs of fluid overload (e.g.shortness of breath, edema). IV hydration rate should be reduced in patients with underlying heart and kidney disease to minimize the risk of symptomatic fluid overload. It is important to note that the routine use of loop diuretics such as furosemide is not recommended due to volume depletion and the development of electrolyte abnormalities. The use of furosemide should be reserved for patients who develop signs of fluid overload while receiving IV hydration (69).

### 4.2 Calcitonin

Calcitonin is a potent hypocalcemic hormone produced by the C-cells of the thyroid (70). Pharmacologic doses of calcitonin reduce serum calcium concentration by increasing renal calcium excretion and, more importantly, by reducing bone resorption by interfering with OC function (71). In addition, calcitonin inhibits the osteoclastogenic effects of the NF-kB ligand (RANKL) receptor activator. It should be administered intramuscularly or subcutaneously (72). Calcitonin is safe and adverse effects are not expected except for a hypersensitivity reaction with mild nausea. The starting dose is 4 units/kg (73). Serum calcium is repeated after 4 to 6 hours. If a hypocalcemic response is noted, the patient is calcitonin sensitive and calcitonin may be repeated every 12 hours for a total of 24 to 48 hours. If the response is unsatisfactory, the dose may be increased to 8 units/kg every 6 to 12 hours (total treatment duration 24 to 48 hours) (74). Although a relatively weak agent, it works rapidly and lowers serum calcium concentrations by a maximum of 1 to 2 mg/dL (0.3 to 0.5 mmol/L) starting within 4 to 6 hours. The efficacy of calcitonin is limited to the first 48 hours, even with repeated doses; this is probably due to the development of tachyphylaxis due to receptor downregulation (75). Due to its limited duration of action, calcitonin is more beneficial in symptomatic patients with calcium >14 mg/dL (3.5 mmol/L) when combined with hydration and bisphosphonates (or denosumab in bisphosphonate-resistant patients).

### 4.3 Reducing bone resorption

Bisphosphonates inhibit osteoclastic bone resorption by binding to hydroxyapatite binding sites on bone surfaces (76). When OCs begin to resorb bisphosphonate-impregnated bone, the bisphosphonate released during resorption impairs the OCs’ ability to form folded edges, adhere to the bone surface, and produce protons necessary for sustained bone resorption. Bisphosphonates also reduce their activity by reducing OC progenitor development and recruitment and promoting OC apoptosis (77, 78).

In addition to their inhibitory effects on OCs, bisphosphonates appear to have a beneficial effect on osteoblasts. The mechanism of this effect has been attributed to connexin 43, a gap junction protein that facilitates the activation of protein kinases. However, this anti-apoptotic effect probably does not contribute significantly to the anti-osteoporotic efficacy of bisphosphonates above their potent antiresorptive effects (79).

Bisphosphonates should be given within 48 hours of diagnosis at the latest because they take approximately 2 to 4 days to take effect. Pamidronate is given as 60 to 90 mg IV over 4 to 24 hours (80). Zoledronic acid is given as 4 mg IV over 15 to 30 minutes (81). One of the serious adverse effects of bisphosphonates is nephrotoxicity (82). In patients presenting with abnormal renal function due to underlying renal disease or hypercalcemia, the benefit of treatment should be reviewed and the dose should be reduced if necessary. In addition to bisphosphonate therapy, adequate hydration can help maintain kidney function. For refractory hypercalcemia, retreatment with zoledronic acid may be considered, but a second dose may be administered as soon as 7 days after the first treatment (83). Renal function should be carefully monitored with serum creatinine level before additional doses of zoledronic acid are given. Recommended dose reduction based on creatinine clearance is follows: GFR >60 mL/min, 4 mg; GFR 50 to 60 mL/min, 3.5 mg; GFR 40 to 49 mL/min, 3.3 mg; and GFR 30 to 39 mL/min, 3.0 mg (61).

The most common adverse effect of bisphosphonates is related to impaired renal function. We have already mentioned the adjustment required for this. Other common adverse effects include bone pain and flu symptoms during the first 1 to 2 days after the infusion. Osteonecrosis of the jaw, which is very rare but very important for the patient’s quality of life, can be seen in patients who receive high-dose and long-term treatment, those who have invasive dental procedures during treatment, and patients with poor oral care (84, 85).

### 4.4 Glucocorticoids

Glucocorticoids help lower serum calcium levels by several mechanisms. Glucocorticoids can suppress the synthesis of extrarenal calcitriol through activated mononuclear cells in the lung and lymph nodes. Namely, by inhibiting 1-alpha hydroxylase, they inhibit 1,25(OH)2 D synthesis and thus calcium absorption from the intestine (86). In addition, they also have inhibitory properties on cytokines released directly from tumor cells, which inhibit osteoclastic bone resorption that will occur with these cytokines (87). Glucocorticoids are usually given as 200 to 400 mg/day of hydrocortisone for 3 to 4 days followed by 10 to 20 mg/day of prednisone for 7 days. Treatment should be continued for a maximum of 10 days and should not be continued if hypercalcemia does not respond (30).

### 4.5 Denosumab

Denosumab is a humanized monoclonal antibody that inhibits the binding of RANKL with its receptor RANK (88). It is the agent used in the second-line treatment of patients with bisphosphonate-resistant malignancy hypercalcemia. In a study in which 120 mg subcutaneous denosumab every 4 weeks was compared with 4 mg IV zoledronic acid intravenously every 4 weeks, it was reported that denosumab was more effective in preventing malignant hypercalcemia in patients with metastatic bone disease (89). This study showed that denosumab delayed the first episode of malignancy-associated hypercalcemia (hazard ratio [HR] 0.63)and also reduced the risk of developing recurrent hypercalcemia by 52%. Compared with 40% of the zoledronic acid group, only 31% of those receiving denosumab developed hypercalcemia (90).

The adverse-effect profile of denosumab is also different from that of bisphosphonates. Denosumab, unlike bisphosphonates, is not excreted by the kidney and, as a result, there is no restriction on its use in patients with chronic kidney disease or a decrease in GFR for any reason, for whom bisphosphonates are used with caution or contraindicated (91). Given that pharmacokinetics and pharmacodynamics are not affected by renal status, renal adjustment has not been reported to be necessary. However, because it is a more potent agent, the risk of hypocalcemia is higher in bisphosphonates (92). Careful monitoring of serum calcium levels is required because this risk is seen to be higher in patients with renal failure. Denosumab has also been reported to be effective in cases of parathyroid carcinoma resistant to cinacalcet and IV bisphosphonates (93, 94). Denosumab cesassion may lead to a rapid increase in the concentrations of bone turnover markers. Usually, this markers elevated to above pre-treatment levels (95). This fenomenon also associated with a decline in bone mineral density at skeletal sites and it described as “rebound phenomenon” (96). The underlying mechanism of this phenomenon is thought to be increased RANKL due to discontinuation of antiresorptive agent. Abnormally increased RANKL expression is lead to a mass increase in osteoclastogenesis by premature osteoclasts accumulated during RANKL inhibition (95). For prevention fracture risk and hypercalcemia, bifosfonates could be preferred as subsequent treatment in patients discontinuing denosumab (97).

### 4.6 Cinacalcet

Cinacalcet directly decreases PTH levels by increasing the sensitivity of the calcium-sensing receptor to extracellular calcium, thus leading to a decrease in serum calcium levels (95). The drug is approved for use in tertiary and secondary hyperparathyroidism and refractory parathyroid carcinoma. Parathyroid carcinoma is the only malignancyfor which cinacalcet is approved (96).

### 4.7 Gallium nitrate

Gallium nitrate is thought to exert its hypocalcemic effect by inhibiting calcium absorption from bone (97). Gallium nitrate is localized where bone remodeling occurs and inhibits osteoclast activity. Compared with IV pamidronate, gallium appeared to have generally similar calcium-lowering effects, although it was more successful than pamidronate in epidermoid tumors (98). Gallium nitrate was found to be more potent in a study compared with calcitonin (99). It has also been shown that gallium is effective in tamoxifen-induced hypercalcemia and provides normocalcemia while patients continue tamoxifen treatment (100). The recommended dose is 100 to 200 mg/m^2^ IV over 24 hours for 5 days (101). It is well tolerated and does not show significant nephrotoxicity, but is not currently FDA approved. Gallium nitrate was removed from the US market in 2012

### 4.8 Dialysis

Dialysis is the method used for the treatment of patients in whom optimal hydration cannot be achieved safely due to heart or kidney failure or who have hypercalcemia unresponsive to other treatments (6). It can also be performed as an emergency treatment in patients who develop arrhythmia due to severe hypercalcemia (102). The dialysis solution used for this purpose consists of a calcium-free acetate solution or a dialysate with a very low calcium level (103).

## 5 Conclusion

Hypercalcemia due to malignancy is a very important issue in terms of both being the most common cause of hypercalcemia and affecting the prognosis of patients with cancer. Hypercalcemia can present with a wide range of findings from mild symptoms to life-threatening symptoms. Hematology,oncology, internal medicine,and palliative care specialists should have sufficient knowledge about the diagnosis and management of hypercalcemia.

## Author contributions

All authors meet the ICMJE authorship criteria and were involved in the whole writing process.
